# Novel Antioxidant Peptides from Pearl Shell Meat Hydrolysate and Their Antioxidant Activity Mechanism

**DOI:** 10.3390/molecules28020864

**Published:** 2023-01-15

**Authors:** Pantian Huang, Jianyin Miao, Jialing Li, Yingkun Li, Xianghua Wang, Yan Yu, Yong Cao

**Affiliations:** 1 Guangdong Provincial Key Laboratory of Nutraceuticals and Functional Foods, College of Food Science, South China Agricultural University, Guangzhou 510642, China; 2 Beihai Black Pearl Marine Biotechnology Co., Ltd., Beihai 536000, China

**Keywords:** pearl shell meat, antioxidant peptides, purification and identification, cellular antioxidant activity, cytoprotective effect, activity mechanism

## Abstract

Free radicals are associated with aging and many diseases. Antioxidant peptides with good antioxidant activity and absorbability are one of the hotspots in antioxidant researches. In our study, pearl shell (*Pinctada martensii*) meat hydrolysate was purified, and after identification by proteomics, six novel antioxidant peptides SPSSS, SGTAV, TGVAS, GGSIT, NSVAA, and GGSLT were screened by bioinformatics analysis. The antioxidant peptides exhibited good cellular antioxidant activity (CAA) and the CAA of SGTAV (EC_50_: 0.009 mg/mL) and SPSSS (EC_50_: 0.027 mg/mL) were better than that of positive control GSH (EC_50_: 0.030 mg/mL). In the AAPH-induced oxidative damage models, the antioxidant peptides significantly increased the viability of HepG2 cells, and the cell viability of SGTAV, SPSSS, and NAVAA were significantly restored from 79.41% to 107.43% and from 101.09% and 100.09%, respectively. In terms of antioxidant mechanism by molecular docking, SGTAV, SPSSS, and NAVAA could tightly bind to free radicals (DPPH and ABTS), antioxidant enzymes (CAT and SOD), and antioxidant channel protein (Keap1), suggesting that the antioxidant peptides had multiple antioxidant activities and had structure–activity linkages. This study suggests that the antioxidant peptides above are expected to become new natural materials for functional food industries, which contribute to the high-value applications of pearl shell meat.

## 1. Introduction

Any organism that breathes aerobically produces free radicals [1]. The antioxidant defense system plays an important role in scavenging reactive oxygen species (ROS) and preventing cell damage. However, excessive production of ROS will lead to oxidative damage and initiate the oxidation of biomolecules such as DNA, RNA, and membrane lipids, which may further cause disorders of the body, leading to premature aging or diseases such as cancer, atherosclerosis, diabetes, and cardiovascular diseases [2]. Therefore, adequate intake of antioxidants is required to prevent or slow down the oxidative stress caused by ROS and free radicals. Although antioxidants such as butyl hydroxytoluene (BHT), tert-butylhydroquinone (TBHQ), and butyl hydroxyanisole (BHA) can reduce the level of free radicals in food, they are potentially toxic to human health [3,4]. In recent years, antioxidants extracted from natural sources, including animals, plants, or microorganisms such as polyphenols [5], polysaccharides [6], and peptides [7] with antioxidant properties, have become a research hotspot.

Protein hydrolysates with antioxidant activity are a kind of popular antioxidants that have both biological activity and recognized nutritional properties [8]. More and more studies have shown that bioactive peptides with antioxidant activity can be prepared by enzymatic hydrolysis of foodborne proteins [3]. Jayaprakash et al. [9] prepared bioactive peptides with antioxidant activity by enzymatic hydrolysis of mussels. Je et al. [10] obtained an antioxidant peptide (VKAGFAWTANQQLS) by enzymatic hydrolysis of tuna backbone protein. Chang-Bum et al. [2] found a hepatoprotective antioxidant peptide (FLNEFLHV) from the pectoral fin protein of salmon byproduct. Shang et al. [11] isolated antioxidant peptides from sea urchin gonads. These studies exhibited that it is feasible to extract antioxidant peptides from foodborne proteins. Meanwhile, bioactive peptides have the advantages of high safety and stable properties [12]. Therefore, it is of great significance to develop antioxidant peptides and conduct in-depth study on the related mechanisms of antioxidation.

*Pinctada martensii*, also known as pearl shell, is the main species of seawater pearl aquaculture in China. A large amount of pearl shell meat is produced after pearl picking every year. However, due to the lack of deep processing, most pearl shellfish meat is discarded as a byproduct, which not only wastes a great deal of resources but also causes environmental pollution. Pearl shellfish meat is a good protein resource, and the protein content of its dry weight is as high as 74.9% [13]. Traditional Chinese medicine believes that pearls have good health care and medicinal value. Modern studies have shown that the peptides prepared from pearl shell meat have a variety of biological activities, such as antioxidation [14], skin healing [15], promotion of osteoblast proliferation [16], and so on. At present, there are few reports on the mechanism of antioxidant peptides in pearl shell meat, so we undertook an in-depth study on the antioxidant peptides of pearl shellfish meat.

In the previous study, we determined the optimal process for the preparation of antioxidant hydrolysates from pearl shell meat [17]. In this study, the antioxidant hydrolysate of pearl shell meat was isolated and purified, and the peptide map composition of peptides was identified. Then, the potential antioxidant peptides were screened by bioinformatics technology, and the cellular antioxidant activity and oxidative damage protection of antioxidant peptides were evaluated. Finally, the interaction between antioxidant peptides and free radicals (ABTS and DPPH), antioxidant enzymes (CAT and SOD), and channel protein Keap1 was explored by molecular docking technique, and the antioxidant activity mechanism of antioxidant peptides was analyzed comprehensively. Through the above studies, efficient antioxidant peptides were screened in order to provide raw materials for the development of functional food, cosmetics, and medical products so as to realize the high-value utilization of pearl shell meat.

## 2. Results and Discussion

### 2.1. Purification and Antioxidant Activities of Antioxidant Peptides

#### 2.1.1. Separation of the Pearl Shell Meat Hydrolysate by Ultrafiltration

**Figure 1:**
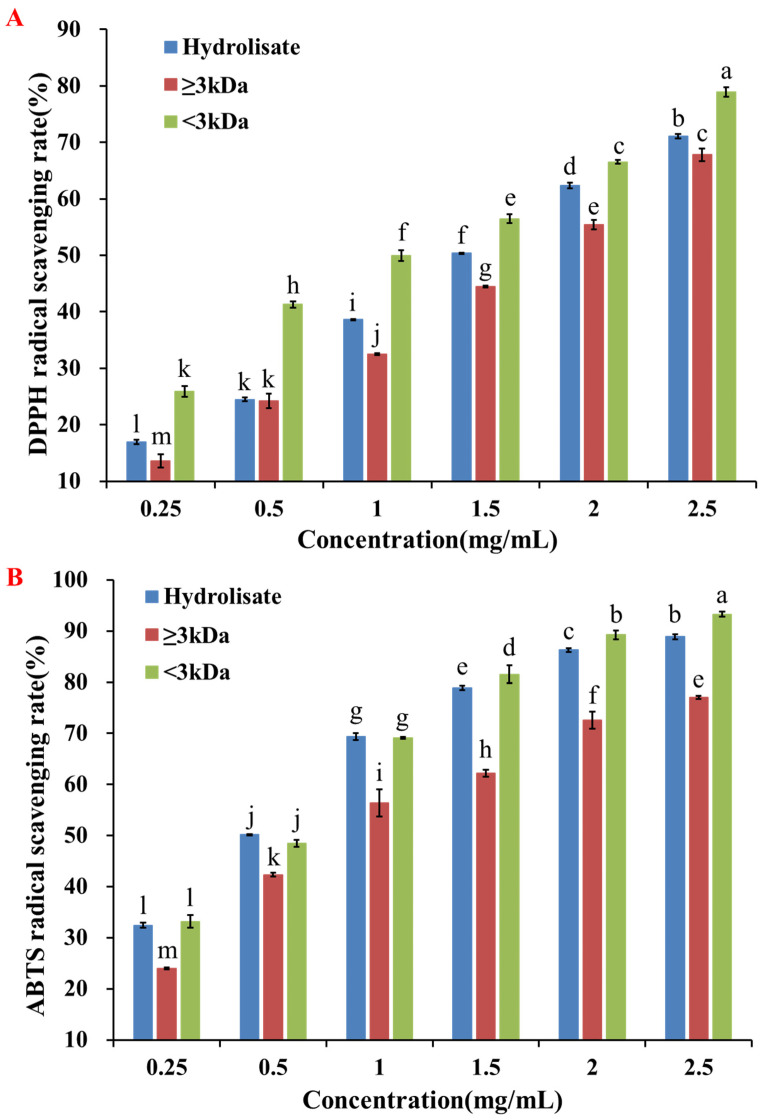
Separation of the pearl shell meat hydrolysate by ultrafiltration and the evaluation of antioxidant activity. (**A**) DPPH radical scavenging rate of the hydrolysate and ultrafiltration fractions (≥3 kDa and <3 kDa). (**B**) ABTS radical scavenging rate of the hydrolysate and ultrafiltration fractions (≥3 kDa and <3 kDa). Different letters (a–m) indicated that the difference between the data was statistically significant (*p* < 0.05).

The antioxidant hydrolysate of pearl shellfish meat was prepared according to the previously determined process parameters [17]. The hydrolysate was separated by a 3 kDa ultrafiltration membrane to obtain two fractions: ≥3 kDa and <3 kDa. The fraction <3 kDa showed stronger free radical scavenging activity than the hydrolysate and the fraction ≥3 kDa (*p* < 0.05) (Figure 1). At 1 mg/mL, scavenging rates of DPPH and ABTS free radicals of the fraction <3 kDa were 49.97% and 69.15%, respectively. Peptides with low molecular weight usually have stronger antioxidant activities [18]; hence, the fraction <3 kDa was gathered and further purified.

#### 2.1.2. Purification of the Fraction <3 kDa by RP-HPLC

**Figure 2:**
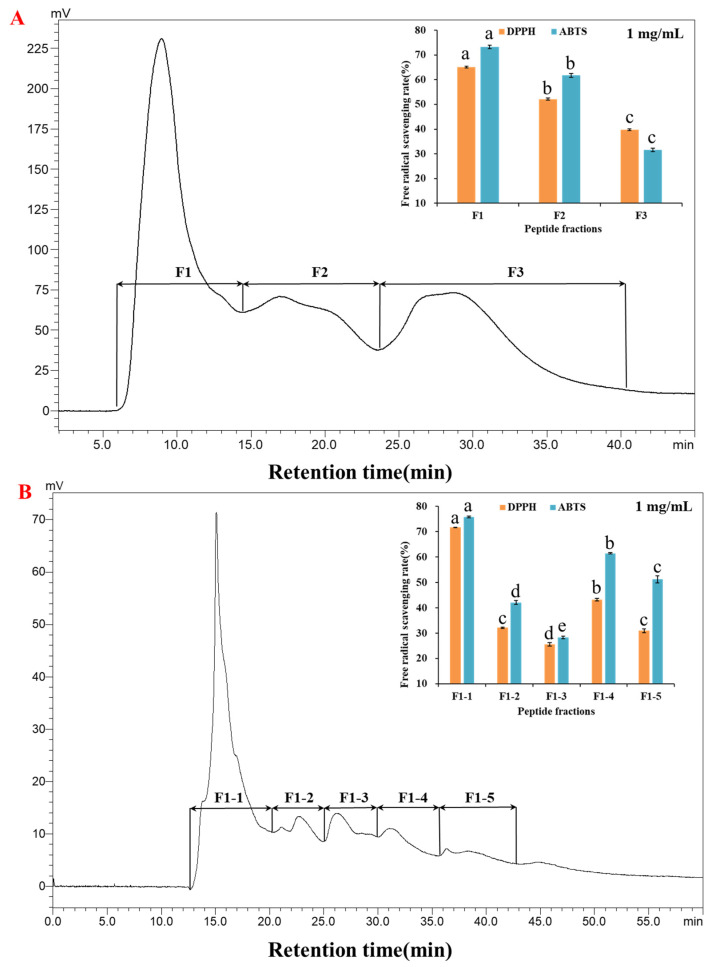
Purification of the <3 kDa by RP-HPLC and the evaluation of antioxidant activity. (**A**) Primary purification chromatogram of <3 kDa fraction and the antioxidant activity of each purified fraction F1~F3. (**B**) Secondary purification chromatogram of fraction F1 and the antioxidant activity of each purified fraction F1-1~F1-5. Different letters (a–e) indicated that the difference between the data was statistically significant (*p* < 0.05).

In the primary purification, the fraction <3 kDa was separated by RP-HPLC, and three peaks were identified as fractions F1~F3. As shown in Figure 2A, fraction F1 with highest response value had the best scavenging rates of DPPH and ABTS free radicals, which were 65.09% and 73.24% at 1 mg/mL, respectively. In the secondary purification, fraction F1 was separated, and five peaks appeared as fractions F1-1~F1-5. Fraction F1-1 had the best scavenging rates of DPPH and ABTS free radicals (Figure 2B), which were 71.68% and 75.82% at 1 mg/mL, respectively. Therefore, the fraction F1-1 was further analyzed by LC-MS/MS to determine the peptide composition.

### 2.2. Identification of Fraction F1-1 and Bioinformatics Analysis

Fraction F1-1 was identified by proteomics, and 12 potential antioxidant peptides were obtained. The amino acid composition and bioinformatics analysis of peptides are shown in Table 1. Studies have shown that hydrophobic amino acids of peptides were a key factor to scavenge free radicals [19]. Peptides with stronger hydrophobicity would have better antioxidant potential [20]. All 12 peptides were non-toxic and completely resistant to the simulated digestion of pepsin and trypsin, with hydrophobicity in the range of 7.95–9.88 Kcal/mol. Among them, the six highly hydrophobic peptides SPSSS, SGTAV, TGVAS and GGSIT, NSVAA, and GGSLT were screened, and the secondary map is shown in Figure S1. Moreover, they contained hydrophobic amino acids (such as Pro, Gly, Ala, Val, Ile, and Leu), indicating that the six peptides had a good basis for antioxidant substances. To evaluate the antioxidant capacity, six peptides were synthesized, and their cellular antioxidant capacities were investigated.

### 2.3. Cellular Antioxidant Activity of Antioxidant Peptides

#### 2.3.1. Cell Viability of HepG2 Cells

**Figure 3:**
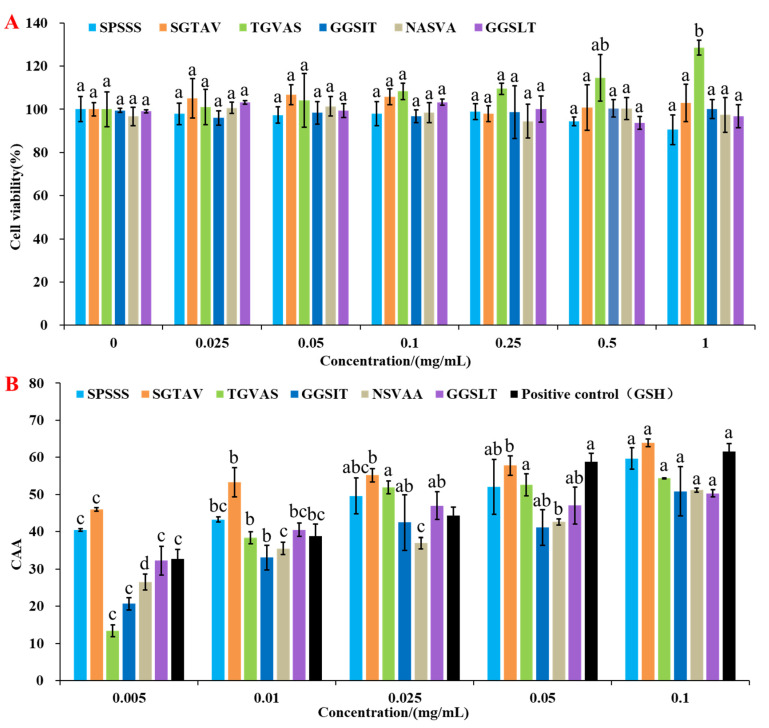
Evaluation of antioxidant activity of antioxidant peptides SPSSS, SGTAV, TGVAS, GGSIT, NSVAA, and GGSLT on HepG2 cells. (**A**) Effect of antioxidant peptides on HepG2 cells viability. (**B**) CAA unit of antioxidant peptides and positive control (GSH). Different letters (a–d) indicated significant differences among the various concentrations (*p* < 0.05) of each sample.

The cell viability of HepG2 cells treated with antioxidant peptides SPSSS, SGTAV, TGVAS, GGSIT, NSVAA, and GGSLT were all above 90% in the concentration range of 0.025~1 mg/mL, indicating that they had no toxic effect on cells [21] (Figure 3A). 

#### 2.3.2. Cellular Antioxidant Activity

The principle of CAA is that intracellular lipase degrades the fluorescent probe DCFH-DA to DCFH, which is oxidized to fluorescent DCF by intracellular free radicals under AAPH induction [22], and the antioxidant peptide can protect the probe from oxidation. The fluorescence profiles of probe oxidation under antioxidant peptides protection were shown in Figure S2. The CAA units of the six antioxidant peptides and the positive control (GSH) are shown in Figure 3B, and the antioxidant peptides all had intracellular antioxidant activities in a concentration-dependent manner. In related reports, the EC_50_ value of a pine nut peptide QDHCH was 0.13 mg/mL [23], and the EC_50_ value of corn-derived antioxidant peptides (CPF1) was 2.85 mg/mL [24]. In our study, the EC_50_ values of the six antioxidant peptides ranged from 0.009 to 0.095 mg/mL (Table 2), which were lower than relevant reports, indicating that they all had good antioxidant activity. The EC_50_ value of SGTAV and SPSSS was 0.009 and 0.027 mg/mL, respectively, which were lower than 0.030 mg/mL of positive control (GSH), indicating that the CAA of SGTAV and SPSSS were better than that of the positive control (GSH). The cellular oxidative damage protection of six antioxidant peptides was evaluated in the next step.

### 2.4. Cytoprotective Effect of Antioxidant Peptides against AAPH-Induced Cell Damage

#### 2.4.1. Effect of Cell Viability Treated by AAPH

**Figure 4:**
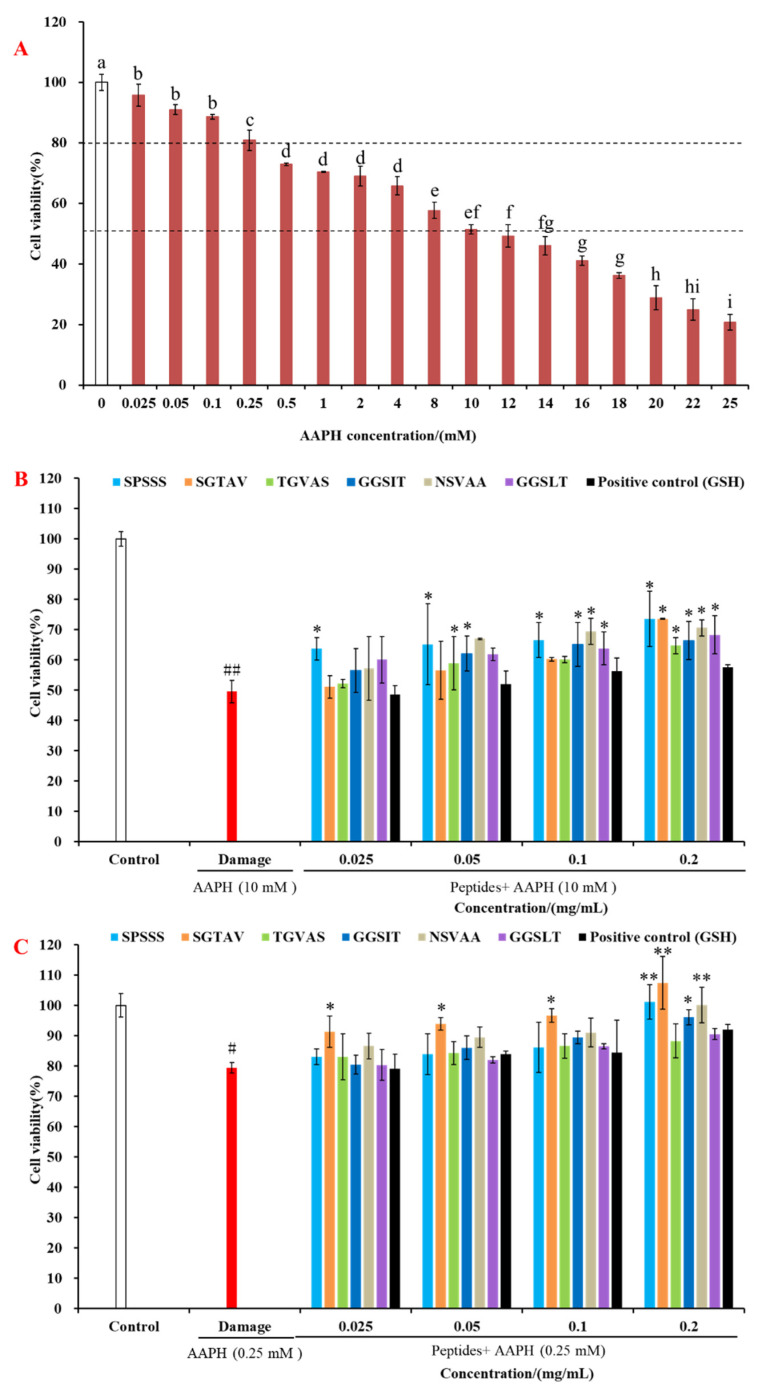
Cytoprotective effects of antioxidant peptides SPSSS, SGTAV, TGVAS, GGSIT, NSVAA, and GGSLT and positive control (GSH) against AAPH-induced stress injury in HepG2 cells. (**A**) Effects of different concentrations of AAPH on the cell viability. (**B**) Protective effect of antioxidant peptide in the 50% cell viability model. (**C**) Protective effect of antioxidant peptide in the 80% cell viability model. Different letters (a-i) indicated that the difference between the data was statistically significant (*p* < 0.05). ## indicated *p* < 0.01 compared with the control group. # indicated *p* < 0.05 compared with the control group. ** indicated *p* < 0.01 compared with the AAPH damage group. * indicated *p* < 0.05 compared with the AAPH damage group.

As shown in Figure 4A, the cell viability of the AAPH damage group was lower than that of the control group and showed a dose-dependent relationship. Considering that the intensity of oxidative damage is often different in actual situations, the damage models with cell viability of 80% and 50% [12,25] were constructed by using 10 mM and 0.25 mM AAPH, respectively, and the cytoprotective effects of antioxidant peptides with different degrees of oxidative damage were compared on this basis. 

#### 2.4.2. Cytoprotective Effect of Antioxidant Peptides

Our study evaluated the protective effects of antioxidant peptides on two AAPH-induced oxidative damage models with cell viability of 50% (Figure 4B) and 80% (Figure 4C). Compared with the control group, the AAPH damage group could significantly reduce the cell viability (*p* < 0.05), indicating that the cell oxidative damage model was successfully established. In the 50% cell viability model, the cell viability treated by the six antioxidant peptides SPSSS, SGTAV, TGVAS, GGSIT, NSVAA, and GGSLT at 0.2 mg/mL increased from 49.51% to 73.52%, 73.61%, 64.69%, 66.35%, 70.55%, and 68.25%, respectively, which were all better than 57.60% of the positive control group (GSH). In the 80% cell viability model, the cell viability treated by antioxidant peptides SPSSS, SGTAV, GGSIT, and NSVAA at 0.2 mg/mL increased from 79.41% to 101.09%, 107.43%, 96.06%, and 100.09%, respectively, which were better than 92.08% of the positive control group (GSH). Among them, antioxidant peptides SPSSS, SGTAV, and NSVAA all restored the injured cell viability to the level of normal cells, indicating that their cytoprotective effects were particularly prominent. Therefore, the effects of SPSSS, SGTAV, and NSVAA on the intracellular antioxidant enzymes CAT and SOD in HepG2 cells were further investigated in the following test.

#### 2.4.3. Effect of Antioxidant Peptides on CAT and SOD 

**Figure 5:**
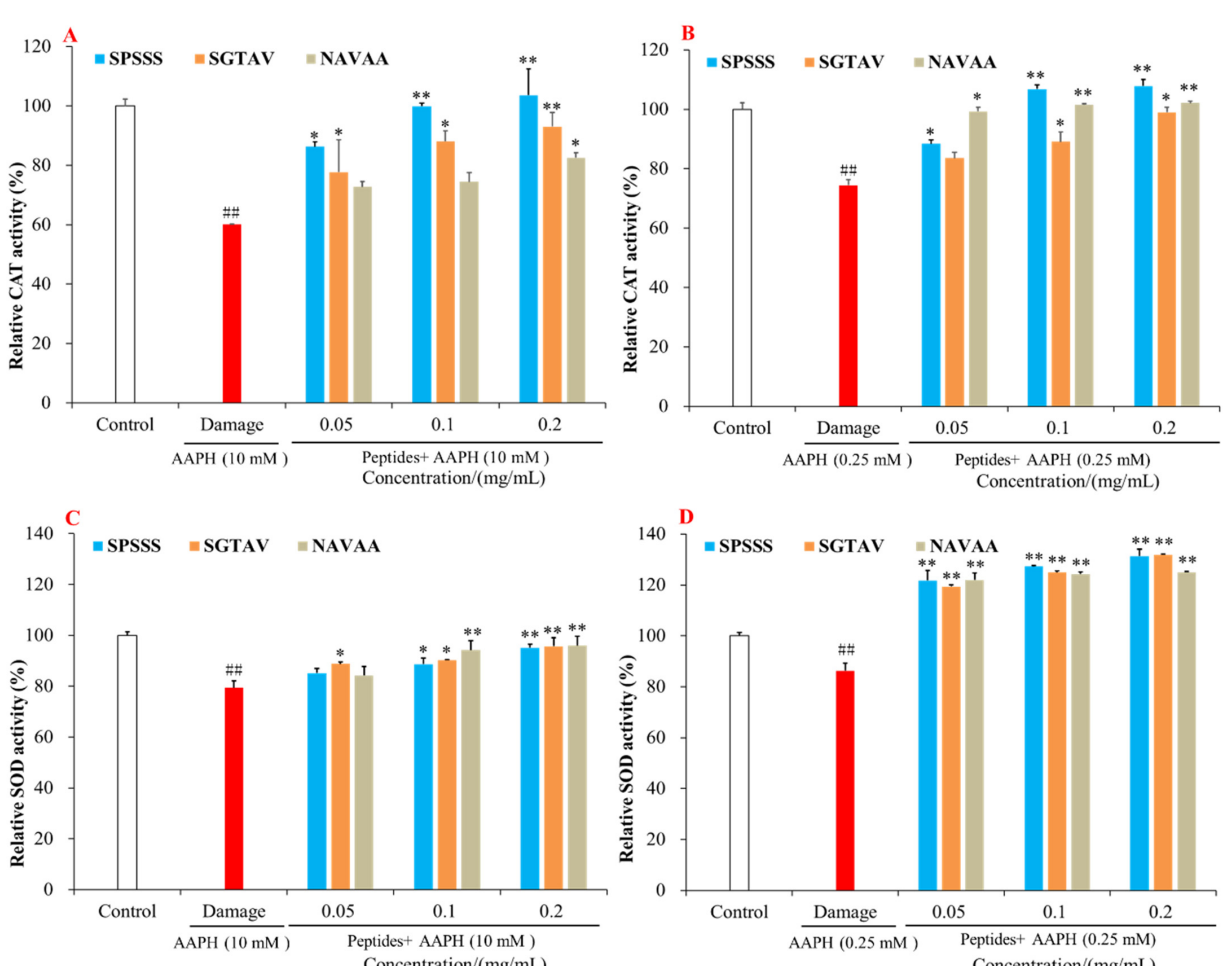
Effect of antioxidant peptides SPSSS, SGTAV, and NSVAA on antioxidant enzymes in AAPH-induced damage cells. (**A**) Effect of antioxidant peptides on CAT in the 50% cell viability model. (**B**) Effects of antioxidant peptides on CAT in the 80% cell viability model. (**C**) Effects of antioxidant peptides effect on SOD in the 50% cell viability model. (**D**) Effects of antioxidant peptides on SOD in the 80% cell viability model. ## indicated *p* < 0.01 compared with the control group. ** indicated *p* < 0.01 compared with the AAPH damage group. * indicated *p* < 0.05 compared with the AAPH damage group.

Antioxidant enzymes can reduce the accumulation of oxygen free radicals in the body and protect cells from oxidative damage [12,26]. By measuring the activities of antioxidant enzymes CAT and SOD, the effects of antioxidant peptides SPSSS, SGTAV, and NSVAA on oxidative damage of HepG2 cells were explored, and the possible mechanism of antioxidant peptides protecting cells was further analyzed. As shown in Figure 5, the contents of SOD and CAT in the AAPH damage group were extremely significantly lower than those in the control group (*p* < 0.01). Consistent with relevant reports, oxidative damage by AAPH results in decreased activities of antioxidant enzymes in HepG2 cells [12]. In the 50% cell viability model, antioxidant peptides SPSSS, SGTAV, and NSVAA at 0.2 mg/mL increased the CAT activity from 60.10% to 103.62%, 93.06%, and 82.53%, respectively, and increased the SOD activity from 79.44% to 95.12%, 95.70%, and 95.96%, respectively. In the 80% cell viability model, antioxidant peptides SPSSS, SGTAV, and NSVAA at 0.2 mg/mL increased the CAT activity from 74.45% to 107.88%, 98.96%, and 102.04%, respectively, and increased the SOD activity from 86.25% to 131.86%, 131.24%, and 125.07%, respectively. Among them, the antioxidant peptide SPSSS had the best effect on enhancing CAT activity, and the CAT activity returned to the normal cell level. Meanwhile, antioxidant peptides SPSSS, SGTAV, and NSVAA could promote the activity of SOD in cells to the normal level. The results showed that SPSSS, SGTAV, and NSVAA could enhance the activities of antioxidant enzymes in AAPH-damaged cells and had good antioxidant effects.

### 2.5. Molecular Docking Simulation of Antioxidant Mechanisms

Molecular docking is a commonly used computer biotechnology technique in functional active ingredient screening, based on the principle of predicting binding conformation and binding affinity through simulated binding of large molecules (receptors) and small molecules (ligands) [27]. Molecular docking has the advantages of being fast, efficient, and accurate in studying the structure–activity relationships of peptides, which can be applied to the study of antioxidant mechanisms [28]. In this study, the antioxidant peptides SPSSS, SGTAV, and NSVAA exhibited good chemical and cellular antioxidant capacity, but the molecular mechanism was still unclear. Therefore, our study explored the molecular mechanism of the antioxidant peptides by predicting the interaction between antioxidant peptides and free radicals (DPPH and ABTS), antioxidant enzymes (CAT and SOD), and antioxidation-related protein (Keap1). 

#### 2.5.1. Interaction between Antioxidant Peptides and Free Radicals

**Figure 6:**
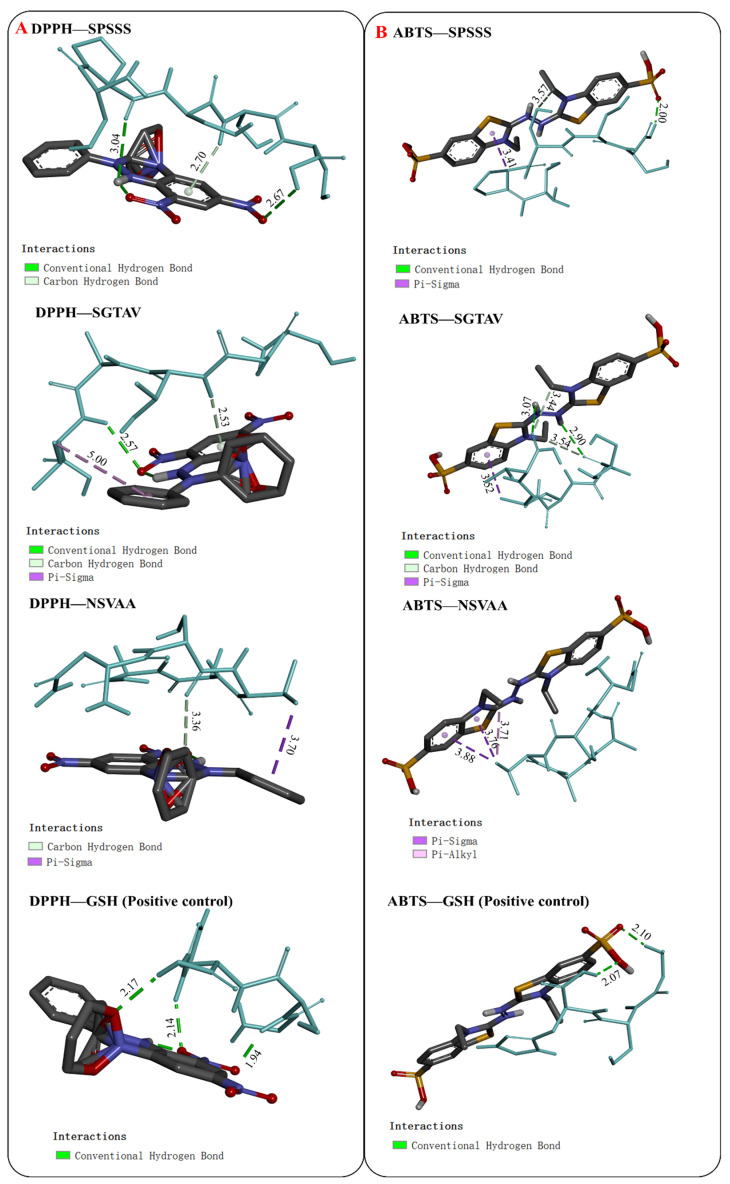
Visualization of molecular docking of antioxidant peptides SPSSS, SGTAV, and NSVAA and positive control (GSH) with free radical DPPH (**A**) and ABTS (**B**).

Molecular docking results with DPPH and ABTS radicals are shown in Figure 6 and Table 3. In the docking with DPPH, the binding energies of SPSSS, SGTAV, and NSVAA were −3.5, −3.3, and −2.8 kcal/mol, respectively, which were equal to or better than the positive control (GSH, −2.8 kcal/mol). DPPH formed hydrogen bonds with SPSSS (SER-4 and SER-5), SGTAV (THR3 and VAL5), and NSVAA (SER2), and DPPH formed hydrophobic interactions with SGTAV (VAL5) and NSVAA (VAL3). In the docking with ABTS, the binding energies of SPSSS, SGTAV, and NSVAA were −2.6, −2.3, and −2.5 kcal/mol, respectively, which were better than that of the positive control (GSH, −2.2 kcal/mol). ABTS formed hydrogen bonds with SPSSS (SER3 and SER5) and SGTAV (SER1 and VAL5), and ABTS formed hydrophobic interactions with SPSSS (PRO2), SGTAV (ALA4), and NSVAA (VAL3). Among the three peptides, SPSSS had the lowest binding energies with both DPPH and ABTS, indicating that SPSSS was bound most closely to free radicals. Previous studies have pointed out that active peptides effectively interact with free radicals to scavenge free radicals [29]. The results showed that the amino acid residues of SPSSS, SGTAV, and NSVAA can form hydrogen bonds or hydrophobic interactions with free radicals, which may play important roles in free radical scavenging activity.

#### 2.5.2. Interaction between Antioxidant Peptides and Antioxidant Enzymes

**Figure 7:**
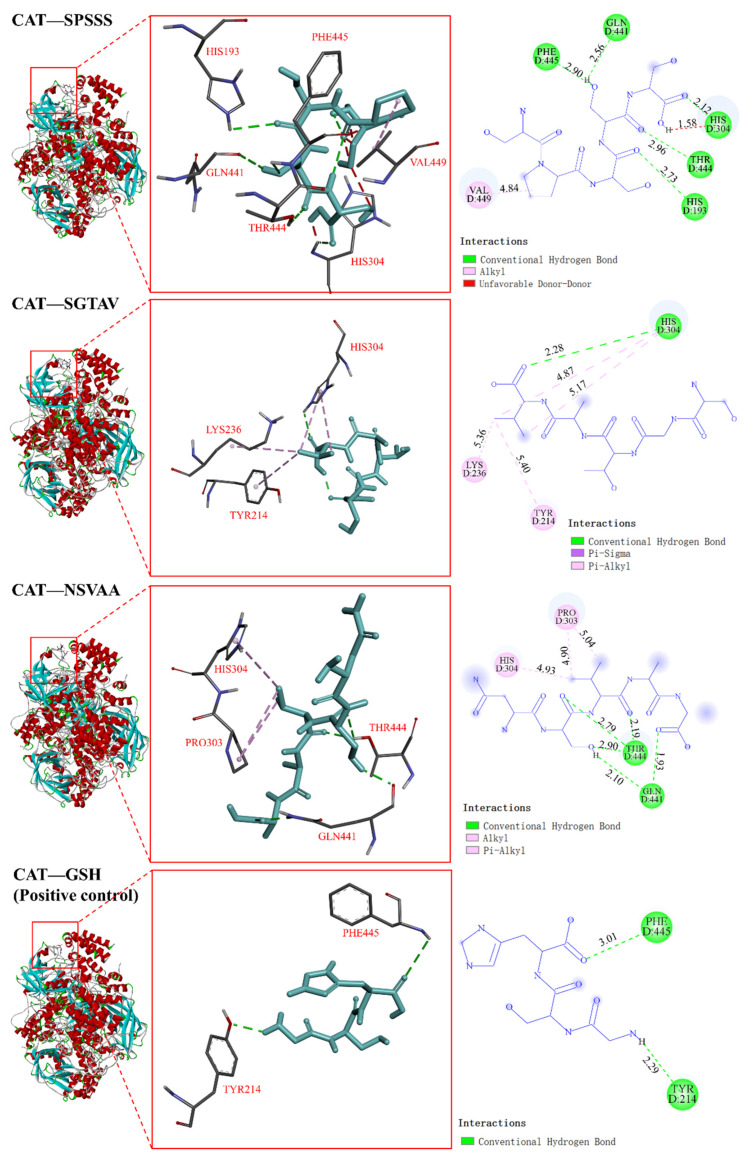
Visualization of molecular docking of antioxidant peptides SPSSS, SGTAV, and NSVAA and positive control (GSH) with CAT.

**Figure 8:**
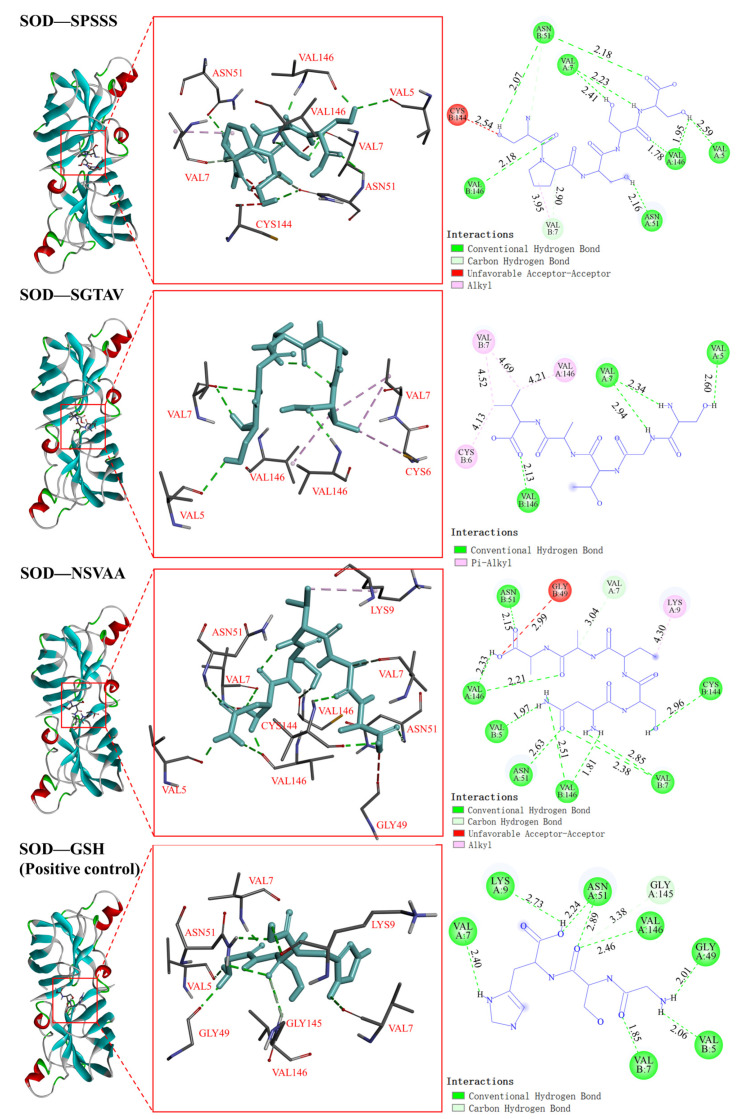
Visualization of molecular docking of antioxidant peptides SPSSS, SGTAV, and NSVAA and positive control (GSH) with SOD.

Antioxidant enzyme activity plays an important role in the control of redox state in various cells, and CAT and SOD are important components of the antioxidant defense system [24]. In the docking with CAT, the binding energy of SPSSS, SGTAV, and NSVAA to CAT was −6.2, −5.4, and −5.5 kcal/mol, respectively, which was better than that of the positive control GSH (−4.9 kcal/mol) (Table 3). As shown in Figure 7, the HIS193, GLN441, HIS304, THR444, and PHE445 residues of CAT formed five hydrogen bonds with SPSSS (SER1, PRO2, SER3, SER4, and SER5), and the VAL449 residue of CAT formed a hydrophobic interaction with SPSSS (PRO2). The HIS304 residue of CAT formed a hydrogen bond with SGTAV (VAL5), and the LYS236, TYR214, and HIS304 residues of CAT formed four hydrophobic interactions with SGTAV (VAL5). The GLN441 and THR444 residues of CAT formed five hydrogen bonds with NSVAA (SER2, VAL3, and ALA5), and the PRO303 and HIS304 residues of CAT formed three hydrophobic interactions with NSVAA (VAL3). Among them, the action sites HIS193, GLN441, THR444, and VAL449 were the same as the CAT binding sites in related reports [30]. In the docking with SOD, the binding energies of SPSSS, SGTAV, and NSVAA to SOD were −5.2, −4.4, and −5.1 kcal/mol, respectively (Table 3). As shown in Figure 8, the VAL5, VAL146, VAL7, and ASN51 residues of SOD formed eleven hydrogen bonds with SPSSS (SER1, PRO2, SER3, SER4, and SER5) and the VAL7 residue of SOD formed a hydrophobic interaction with SPSSS (PRO2). The VAL5, VAL7, and VAL146 residues of SOD formed four hydrogen bonds with SGTAV (SER1, GLY2, THR3, and VAL5), and the CYS6 and VAL164 residue of SOD formed four hydrophobic interactions with SGTAV (VAL5). The CYS144, VAL7, VAL146, ASN51, and VAL5 of SOD formed hydrogen bonds with NSVAA (ASN1, SER2, ALA4, and ALA5), and the LYS9 of SOD formed a hydrophobic interaction with NSVAA (VAL3). Among them, the action sites VAL7, ASN51, and VAL146 were the same as SOD binding sites in related reports [27]. It has been reported that the active substances interact with antioxidant enzymes to protect or enhance enzyme activity and prevent oxidative damage related diseases by regulating antioxidant enzyme system [27,31]. In our research, amino acid residues of SPSSS, SGTAV, and NSVAA were found to bind closely to antioxidant enzymes through hydrogen bonds or hydrophobic interactions, and SPSSS was bound most tightly. The results implied that the antioxidant peptides bound with SOD and CAT, which had the potential to protect or enhance the activity of antioxidant enzymes. Previous studies also demonstrated that antioxidant peptides increased the activities of SOD and CAT in AAPH-induced cells, suggesting that antioxidant peptides may regulate the potential to affect the antioxidant enzyme system.

#### 2.5.3. Interaction between Antioxidant Peptides and Antioxidation-Related Protein (Keap1)

**Figure 9:**
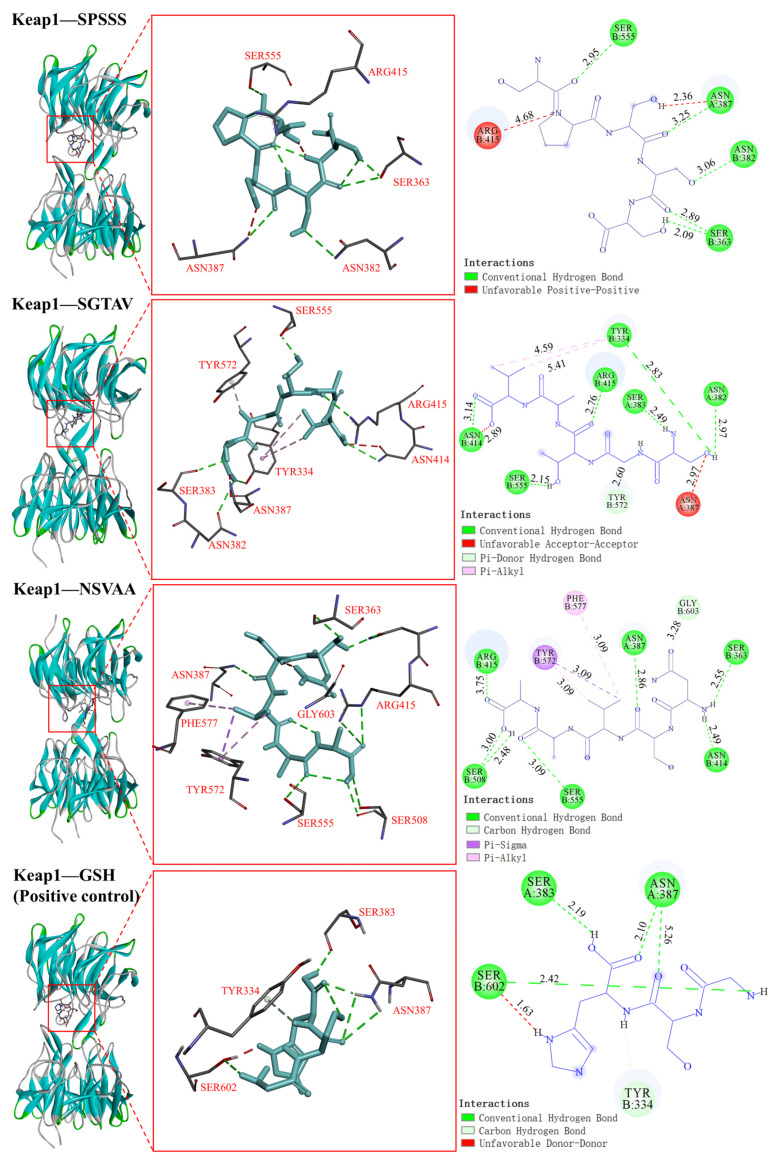
Visualization of molecular docking of antioxidant peptides SPSSS, SGTAV, and NSVAA and positive control (GSH) with Keap1.

Keap1 is a major regulatory factor in cellular oxidative stress response. The Keap1-Nrf2/ARE signaling pathway formed by binding to Nrf2 is an important defense system for the body to resist oxidative damage [32]. It can regulate the expression of antioxidant enzymes (such as SOD and CAT) and is also an important target for the prevention of chronic diseases such as cancer or diabetes, respiratory diseases, and neurodegenerative diseases [33]. Molecular docking results with Keap1 are shown in Table 3. The binding energies of Keap1 binding to SPSSS, SGTAV, and NSVAA were −7.5, −8.0, and −7.6 kcal/mol, respectively, which were also better than the positive control GSH (−6.4 kcal/mol). As shown in Figure 9, the SER363, ASN382, ASN387, and SER555 residues of Keap1 formed five hydrogen bonds with SPSSS (SER1, PRO2, SER3, SER4, and SER5). The TYR334, ASN382, SER383, ASN414, ARG415, SER555, and TYR572 residues of Keap1 formed seven hydrogen bonds with SGTAV (SER1, GLY2, THR3, and VAL5), and the TYR334 residue of Keap1 formed two hydrophobic interactions with SGTAV (VAL5). The SER363, ASN387, ASN414, ARG415, SER508, SER555, and GLY603 residues of Keap1 formed eight hydrogen bonds with NSVAA (ASN1, SER2, ALA4, and ALA4), and the PHE577 and TYR572 residues of Keap1 formed three hydrophobic interactions with NSVAA (VAL3). Among them, the action sites including TYR334, SER363, ASN382, SER383, ASN387, ARG415, TYR572, and PHE577 were the same as the Keap1 binding sites in related reports [34,35]. Tonolo et al. [35] found that milk-derived antioxidant peptides regulate the Keap1-Nrf2 pathway and play an antioxidant role by interacting with key amino acid residues in Keap1 pockets. Studies have shown that the active substance binds to the active site of Keap1 to inhibit the nuclear translocation of Nrf2, thus activating the antioxidant mechanism [34]. Similarly, SPSSS, SGTAV, and NSVAA could spontaneously interact with the active site of Keap1 protein in our study, which may have the potential to alleviate oxidative stress and improve antioxidant capacity.

Based on the docking experiments, we found that the antioxidant peptides SPSSS, SGTAV, and NSVAA all bind tightly to free radicals (DPPP and ABTS), antioxidant enzymes (SOD and CAT), and channel proteins (Keap1). The antioxidant capacity of peptides is highly dependent on amino acid composition and amino acid sequence [7], and the intermolecular interactions are related to the amino acid residue of the peptides [36]. It is reported that hydrophobic amino acids such as Pro, Gly, Ala, and Val have potential antioxidant activity [3]. The three antioxidant peptides SPSSS, SGTAV, and NSVAA contained 20%, 60%, and 60% hydrophobic amino acid residues, respectively, and the hydrophobic amino acid residues of antioxidant peptides easily formed hydrogen bonds with antioxidant targets to help improve antioxidant effect. The Ser residues have antioxidant potential and can effectively form hydrogen bonds with DPPH and ABTS [29]. In our study, the three antioxidant peptides SPSSS, SGTAV, and NSVAA all contained Ser residues, and the Ser residue in each antioxidant peptide formed hydrogen bonds with the receptors, including DPPP, ABTS, SOD, CAT, and Keap1. According to the results of Agrawal et al., repeated identical amino acid residues such as Ser-Ser in SPSSS and Ala-Ala in NSVAA were also beneficial to improve antioxidant activity [36]. Meanwhile, our results implied that Ser, Pro, Ala, and Val residues could be prone to form hydrogen bonds with the receptor, and Pro and Val amino acid residues could be prone to form hydrophobic interactions with the receptor, which may provide some data support for the structure–activity relationship of antioxidant peptides. 

According to the above analysis, the enzymatic hydrolysate of pearl shell meat and the isolated antioxidant peptides all had good antioxidant activities, and the antioxidant peptides SPSSS, SGTAV, and NSVAA may play an antioxidant role by scavenging free radicals, increasing the activity of antioxidant enzymes, and regulating the Keap1-Nrf2 pathway. Although the structure of peptides is relatively stable, the processing stability and gastrointestinal digestion and absorption of peptides need to be considered in the application of peptides. After oral administration, proteins and peptides are hydrolyzed into small peptides by pepsin and trypsin in the gastrointestinal tract, and small peptides are absorbed in the intestinal tract [37]. Zhang et al. [1] found that peptides with <3 kDa have better anti-digestion ability than high-molecular-weight peptides in gastrointestinal simulated digestion. Jang et al. [38] found that sandfish hydrolysates remained more than 79% stable in both acidic and alkaline environments but lost part of their activity in simulated gastrointestinal digestion. Wong et al. [39] studied that antioxidant peptides WAFAPA and MYPGLA had thermal and pH stability, but MYPGLA remained stable, while WAFAPA was partially inactivated during simulated gastrointestinal digestion. Therefore, the processing conditions of peptides should be comprehensively considered according to temperature and pH sensitivities in order to avoid destroying the activity of peptides. Hence, whether the antioxidant peptides of pearl shell meat can maintain good biological activity in gastrointestinal digestion needs to be further studied. If necessary, the embedding technology [40] and the construction of delivery systems [37] for peptides will be used to protect the activity of peptides in gastrointestinal digestion.

## 3. Materials and Methods 

### 3.1. Materials

The pearl shell meat was provided by Beihai Black Pearl Marine Biotechnology Co., Ltd. (Guangxi, China). Neutral protease (10 kU/g) was purchased from Nanning Pangbo Biological Engineering Co., Ltd. (Nanning, China). Glutathione (GSH) was purchased from Shanghai Yuanye Biotechnology Co., LTD. (Shanghai, China). DMEM medium, fetal bovine serum, phosphate buffer solution (PBS), and penicillin-streptomycin-neomycin antibiotic mixture were purchased from Thermo Fisher Scientific (China) Co., Ltd. (Shanghai, China). 3-(4,5-Dimethyl-2-Thiazolyl)-2,5-Diphenyl Tetrazolium Bromide (MTT) was purchased from Labgic Technology Co., Ltd. (Beijing, China). All other reagents used in the study were of analytical grade and commercially available.

### 3.2. Preparation of Pearl Shell Meat Hydrolysate

The preparation process of the pearl shell meat hydrolysate had determined previously [17]. Briefly, the pearl shell meat was stirred into minced meat, and ultra-pure water was added (material–liquid ratio 1: 1, *w/w*). The mixture (final pH 7.25) with neutral protease (enzyme to substrate 0.4%, *w/w*) was maintained at 50 °C for 3 h. After hydrolysis, the mixture was heated at 90 °C for 15 min and then cooled to room temperature. After centrifugation at 4000 r/min for 15 min, the pearl shell meat hydrolysate was obtained and stored at −20 °C for further analysis.

### 3.3. Separation and Purification of Pearl Shell Meat Hydrolysate

#### 3.3.1. Separation of Pearl Shell Meat Hydrolysate by Ultrafiltration

The pearl shell meat hydrolysate was separated by an ultrafiltration membrane (3 kDa). Two fractions (<3 kDa and ≥3 kDa) were obtained, gathered individually, and lyophilized (Alpha 2–4 LD plus, Christ Co., Berlin, Germany) for follow-up study.

#### 3.3.2. Purification of the Highly Antioxidant Fraction by RP-HPLC

The highly antioxidant fraction in ultrafiltration was purified in two steps using a preparative HPLC system (LC-8, Shimadzu, Kyoto, Japan). In the first step, the highly active fraction was loaded onto a well-balanced reverse C18 column (20 mm × 450 mm, 10 μM, Shimadzu) with 3 mL loading volume. The HPLC mobile phase consisted of eluent A (ultra-pure water + 0.1%TFA) and eluent B (methanol + 0.1%TFA). The elution of the first purification step was performed as follows: 1–45 min, 5–10% B; 45–65 min, 10–20% B; 65–75 min, 20–50% B; and 75–85 min, 50–95% B. The flow rate was 10 mL/min, and the detection wavelength was 214 nm. Each peak was collected and freeze dried as an independent fraction, and the activities of fractions were determined. In the second purification step, the fraction with the strongest antioxidant activities was further purified by a reverse C18 column (30 mm × 250 mm, 15 μm, Shimadzu). The elution of the second step was as follows: 1–40 min, 5–10% B and 40–45 min, 10–90% B. The fractions were collected and lyophilized, and the strongest antioxidant active fraction was studied further by proteomics.

### 3.4. Identification of the Antioxidant Peptides Identification by Proteomics

After it was purified in two steps and its activities tracked, the fraction with strongest antioxidant activity was identified by LC-MS/MS, which was dissolved and loaded onto an Easy NLC 1200 system (Thermo Fisher, Waltham, MA, USA) and separated through an Acclaim PepMap C18 column (2 μm, 75 μm × 25 cm, 100 Å). MS data were acquired on a Thermo Fisher Q Exactive mass spectrometer (Thermo Fisher, USA) equipped with a Nano Flex ion source. Data acquisition conditions included ion spray voltage (1.9 KV) and interface heater temperature (275 °C). The PEAKS Studio 8.5 (Bioinformatics Solutions Inc. Waterloo, ON, Canada) software was used to process the mass spectrometry data from the original raw atlas files, and the peptide sequences were retrieved in the *Pinctada Martensii* Species Protein database on Uniprot to determine the primary structure of the peptides.

### 3.5. Screening Potential Antioxidant Peptides by Bioinformatics Analysis

Bioinformatics analysis predicted the physicochemical properties of antioxidant peptides. The potential toxicity of peptides was predicted using ToxinPred (https://webs.iiitd.edu.in/raghava/toxinpred/multi_submit.php (accessed on 20 November 2021)). Peptides were mock-digested in ExPASy peptide cutter (https://web.expasy.org/peptide_cutter/ (accessed on 20 November 2021)) under conditions of pepsin (pH = 1.3 and >2.0) and trypsin, and the lowest cleavage probability was 20% [29]. The hydrophobicity of peptides was calculated by Pepdraw (http://www.pepdraw.com/ (accessed on 20 November 2021)). Non-toxic, anti-digested, and highly hydrophobic peptide sequences would be screened.

### 3.6. Synthesis of Antioxidant Peptides

The screened peptides were synthesized by solid-phase synthesis using Fmoc chemistry principles in Synpeptide Co., Ltd. (Nanjing, China). The peptides were determined by LC-MS to confirm the purity more than 98%.

### 3.7. Evaluation of Antioxidant Capacity In Vitro

#### 3.7.1. DPPH Radical Scavenging Ability Assay

The method was modified according to Yang et al. [41]. DPPH solution (100 µL, 0.2 mM in 95% ethanol) was mixed with 100 µL of sample solution (0.25 to 2 mg/mL) and reacted in the dark for 30 min. The absorbance values were at 517 nm in a microplate reader (Enspire Xenon Light Module, 200 Perkin-Elmer, Beaconsfield, UK). The DPPH scavenging activity was calculated by the Formula (1):

(1) $$\mathrm{DPPH}\;\mathrm{radical}\;\mathrm{scavenging}\;\mathrm{rate}/\%=\left[1-\left(A_{t}-A_{c}\right)/A_{0}\right]\times 100/\%$$

where *A_t_*, *A_c_*, and *A_o_* were the absorbance of the test sample, the control, and the blank group, respectively.

#### 3.7.2. ABTS Radical Scavenging Ability Assay

The method was referenced to Floegel et al. [42] with slight modifications. The ABTS^·+^ working solution including ABTS solution (5 mL, 7 mM) and potassium persulfate solution (88 μL, 140 mM) was maintained in the dark for 12 h and diluted to an absorbance of 0.7 ± 0.02 at the wavelength of 734 nm. Then, the mixture of ABTS^·+^ working solution (100 μL) and sample solution (100 μL, 0.25–2 mg/mL) was incubated in the dark for 10 min, and the absorbance value was measured at 734 nm. The ABTS scavenging activity was calculated by the Formula (2):

(2) $$\mathrm{ABTS}\;\mathrm{radical}\;\mathrm{scavenging}\;\mathrm{rate}/\%=\left[1-\left(A_{t}-A_{c}\right)/A_{0}\right]\times 100/\%$$

where *A_t_*, *A_c_*, and *A_o_* were the absorbance of the test sample, the control, and the blank group, respectively.

### 3.8. Evaluation on Cellular Antioxidant Activity of Antioxidant Peptides

#### 3.8.1. Cell Culture

Obtained from National Collection of Authenticated Cell Cultural (Shanghai, China), HepG2 cells between 20 and 35 passages were grown in medium (DMEM supplemented with 10% fetal bovine serum, 1% antibiotic–antimycotic solution) in a 5% CO_2_ incubator at 37 °C.

#### 3.8.2. Cell Viability Assay

The effect of peptides on the survival rate of HepG2 cells was determined by MTT assay [43]. The HepG2 cells were treated with 100 µL of medium with peptides (0.025–1 mg/mL) after they were planted in 96-well plates (1 × 10^4^ cells/well) and cultured for 24 h. The medium without peptide was set as a control group. After 48 h, the HepG2 cells were treated with MTT solution (100 µL, 0.5 mg/mL) for 4 h. Then, the MTT solution was replaced by DMSO, and the plates were shaken for 10 min to dissolve all blue-violet crystals, which were measured at a wavelength of 490 nm to estimate the cell viability.

#### 3.8.3. Cellular Antioxidant Activity Assay

Cellular antioxidant activity (CAA) was determined according to the method of Wolfe et al. [22]. HepG2 cells were seeded in 96-well plates (6 × 10^4^ cells/well) and cultured for 24 h. DCFH-DA working solution (50 µL, 50 µM) and 50 µL of medium with sample (0.005–0.1 mg/mL) were added in the sample treated group. DCFH-DA working solution (50 µL, 50 µM) and sterile water (50 µL) were added to the control group and the blank group. After incubated for 1 h, the cells were washed with PBS. Then, AAPH working solution (100 µL, 600 µm) was added to the sample treated group and the control group, and fresh medium was added to the blank group. Fluorescence values were determined every 5 min for 1 h at an excitation wavelength of 485 nm and a determination wavelength of 528 nm. Glutathione (GSH) was used as a positive control. The CAA unit was calculated by the area of fluorescence curve (AUC), as shown in Equation (3). The median effective concentration (EC_50_) of the samples was calculated, and Trolox was used as the standard antioxidant [24]. The Trolox equivalent (TEAC µM TE/g peptides) was calculated to represent the CAA value.

(3) $$\mathrm{CAA}=\left[1-\left({\mathrm{AUC}}_{t}-{\mathrm{AUC}}_{o}\right)/\left({\mathrm{AUC}}_{c}-{\mathrm{AUC}}_{o}\right)\right]\times 100$$

where AUC_t_, AUC_c_, and AUC_o_ were the fluorescence curve area of the test sample, the control, and the blank group, respectively.

### 3.9. Evaluation on Cytoprotective Effects of Antioxidant Peptides against AAPH-Induced Cell Damage

#### 3.9.1. AAPH-Induced Cell Damage

HepG2 cells were seeded in 96-well plates (1 × 10^4^ cells/well) and cultured for 48 h. To the AAPH damage group, we added 150 µL of fresh medium containing AAPH (final concentration 0.025–25 mM), and to the control group, we added fresh medium without AAPH. After culturing for 24 h, cell viability was determined by the MTT method.

#### 3.9.2. Cytoprotective Effect of Antioxidant Peptides

HepG2 cells were seeded in 96-well plates (1 × 10^4^ cells/well) and cultured for 24 h. To the sample treated group, we added 150 µL of fresh medium containing samples (0.025–0.2 mg/mL). To the control group and AAPH damage group, we added fresh medium without samples. After treating for 24 h, to the sample treated group and AAPH damage group, we added 50 µL of fresh medium containing AAPH (final concentrations of 0.25 and 10 mM). After culturing for 24 h, cell viability was determined.

#### 3.9.3. Protective Effect of Antioxidant Peptides on CAT and SOD

HepG2 cells were seeded in 6-well plates (2.5 × 10^4^ cells/well) and cultured for 24 h. To the sample treated group was added 2 mL of fresh medium containing samples (0.05–0.2 mg/mL). To the control group and AAPH damage group, we added fresh medium without sample. After treating for 24 h, to the sample treated group and AAPH damage group, we added 0.5 mL of fresh medium containing AAPH (final concentrations of 0.25 and 10 mM). After continuously culturing for 24 h, the cells were collected and crushed, and the activities of antioxidant enzymes in the cells were determined by CAT and SOD kits (Nanjing Jiancheng Bioengineering Institute).

### 3.10. Study on the Activity Mechanism of Antioxidant Peptides

Computer simulation docking was used to explore the intermolecular interactions between antioxidant peptides and free radicals (DPPH and ABTS) [29], antioxidant enzymes (SOD and CAT) [27,44], and channel proteins (Keap1) [28]. Molecular docking was performed using the docking software AutoDockVina under Windows10 system. The 3D structure of antioxidant peptides was designed, and the energy-minimized structure was derived using the drawing software MarvinSketch. The 3D structure of DPPH (CID: 2735032) and ABTS (CID: 5360881) were downloaded from *PubChem* database (https://pubchem.ncbi.nlm.nih.gov/ (accessed on 27 December 2021)). The 3D structures of SOD (PDB ID: 4MCM), CAT (PDB ID: 1DGB), and Keap1 (PDB ID: 4L7B) were downloaded from the *Protein Data Bank* (https://www.rcsb.org/ (accessed on 27 December 2021)). Then, all of the 3D structures were imported into AutoDockTools (ADT) for pretreatment, and search grids were set [27,28,29]. The AutoDockVina docking program was run to simulate the docking of antioxidant peptides with DPPH and ABTS free radicals, select the optimal docking conformation according to the built-in scoring function, and use Discovery Studio Visualizer for visual analysis. AutoDockVina docking program was run to simulate the docking, and the optimal docking conformation was selected according to the built-in scoring function and visualized by Discovery Studio Visualizer.

### 3.11. Statistical Analysis

All experiments were conducted at least in triplicate. Microsoft Excel 2019 was used to analyze the data. One-way analysis of variance (ANOVA) with Tukey’s test in SPSS was used for multiple comparisons. Significant differences were considered statistically significant at *p* < 0.05.

## 4. Conclusions

In our study, pearl shell meat hydrolysate was purified, and six novel peptides, namely, SPSSS, SGTAV, TGVAS, GGSIT, NSVAA, and GGSLT, were screened, and we evaluated the cellular antioxidant activity. The three most effective antioxidant peptides, SPSSS, SGTAV, and NSVAA, were further selected, and we further studied their structure–activity relationship and antioxidant mechanism by molecular simulation. The results exhibited that SPSSS, SGTAV, and NSVAA may play an antioxidant effect by binding to free radicals and interacting with the active sites of antioxidant enzymes and Keap1. Overall, the antioxidant peptides from pearl shellfish meat have the potential to be used as a safe and effective antioxidant in the food or cosmetics industry. Our future research will use multispectral technology or molecular dynamics to study the antioxidant peptides.

## Figures and Tables

**Table 1:** Identification results and peptide characteristics of fraction F1-1.

Peptide	Length	Mass (Da)	−10 lgP	pI	Toxicity	Post-digestive Fragments	Hydrophobicity (Kcal/mol)
SPSSS	5	463.1914	15.40	5.38	None	SPSSS	+9.88
SGTAV	5	433.2173	17.02	5.54	None	SGTAV	+9.80
TGVAS	5	433.2173	15.71	5.36	None	TGVAS	+9.80
GGSIT	5	433.2173	20.65	5.36	None	GGSIT	+9.79
NSVAA	5	460.2281	16.37	5.38	None	NSVAA	+9.75
GGSLT	5	433.2173	20.65	5.36	None	GGSLT	+9.66
ITIGG	5	459.2693	21.13	5.60	None	ITIGG	+8.21
ITTSS	5	507.2540	18.19	5.46	None	ITTSS	+8.20
LTIGG	5	459.2693	21.13	5.60	None	LTIGG	+8.08
ITLGG	5	459.2693	21.13	5.60	None	ITLGG	+8.08
LTTSS	5	507.2540	18.19	5.45	None	LTTSS	+8.07
LTLGG	5	459.2693	21.13	5.60	None	LTLGG	+7.95

**Table 2:** The median effective concentrations (EC_50_) and cellular antioxidant activity (CAA) values of six antioxidant peptides.

	Peptides	EC_50_ (mg/mL)	CAA Value (mM TE/g Peptide)
Antioxidant peptides	SGTAV	0.009	15.072
	SPSSS	0.027	5.130
	TGVAS	0.037	3.704
	GGSLT	0.064	2.152
	GGSIT	0.073	1.874
	NSVAA	0.095	1.443
Positive control	GSH	0.030	4.593

**Table 3:** Molecular docking results of antioxidant peptides with related receptors.

	Peptides	Receptors	Binding Energy (kcal/mol)	Hydrogen Bonds		Hydrophobic Interaction	
				Quantity	Binding Sites	Quantity	Binding Sites
Antioxidant peptides	SPSSS	DPPH	−3.5	3	DPPH	0	DPPH
	SGTAV		−3.3	2	DPPH	1	DPPH
	NSVAA		−2.8	1	DPPH	1	DPPH
Positive control	GSH		−2.8	3	DPPH	0	—
Antioxidant peptides	SPSSS	ABTS	−2.6	2	ABTS	1	ABTS
	SGTAV		−2.3	4	ABTS	1	ABTS
	NSVAA		−2.5	0	—	3	ABTS
Positive control	GSH		−2.2	2	ABTS	0	—
Antioxidant peptides	SPSSS	CAT	−6.2	5	HIS193, GLN441, HIS304, THR444, PHE445	1	VAL449
	SGTAV		−5.4	1	HIS304	4	LYS236, TYR214, HIS304
	NSVAA		−5.5	5	GLN441, THR444	3	PRO303, HIS304
Positive control	GSH		−4.9	2	PHE445, TYR214	0	—
Antioxidant peptides	SPSSS	SOD	−5.2	11	VAL5, VAL146, VAL7, ASN51	1	VAL7
	SGTAV		−4.4	4	VAL5, VAL7, VAL146	4	VAL7, CYS6, VAL164
	NSVAA		−5.1	11	CYS144, VAL7, VAL146, ASN51, VAL5	1	LYS9
Positive control	GSH		−6.7	9	VAL7, VAL5, GLY49, VAL146, GLY145, ASN51, LYS9	0	—
Antioxidant peptides	SPSSS	Keap1	−7.5	5	SER363, ASN382, ASN387, SER555	0	—
	SGTAV		−8.0	7	TYR334, ASN382, SER383, ASN414, ARG415, SER555, TYR572	2	TYR334
	NSVAA		−7.6	8	SER363, ASN387, ASN414, ARG415, SER508, SER555, GLY603	3	PHE577, TYR572
Positive control	GSH		−6.4	5	ASN387, SER383, SER602, TYR334	0	—

## Data Availability

Not applicable.

## Supplementary Materials

The following supporting information can be downloaded at: https://www.mdpi.com/article/10.3390/molecules28020864/s1, Figure S1: Secondary mapping of antioxidant peptides in proteomics. (A) SPSSS; (B) STGAV; (C) TGVAS; (D) GGSIT; (E) NSVAA; (F) GGSLT; Figure S2: Curve of the effect of antioxidant peptides SPSSS, SGTAV, TGVAS, GGSIT, NSVAA, GGSLT and positive control (GSH) on probe fluorescence intensity of HepG2 cells in CAA.

## Sample Availability

Samples of the compounds are not available from the authors.
