# Supplementary material for: Novel Antioxidant Peptides from Pearl Shell Meat Hydrolysate and Their Antioxidant Activity Mechanism
Source: Molecules. 2023 Jan 15;28(2):864. doi: 10.3390/molecules28020864 (PMC9862034; doi:10.3390/molecules28020864)
Supplement: Supplementary file 1 [file molecules-28-00864-s001.zip › molecules-2131582-supplementary.pdf]

Supplementary material

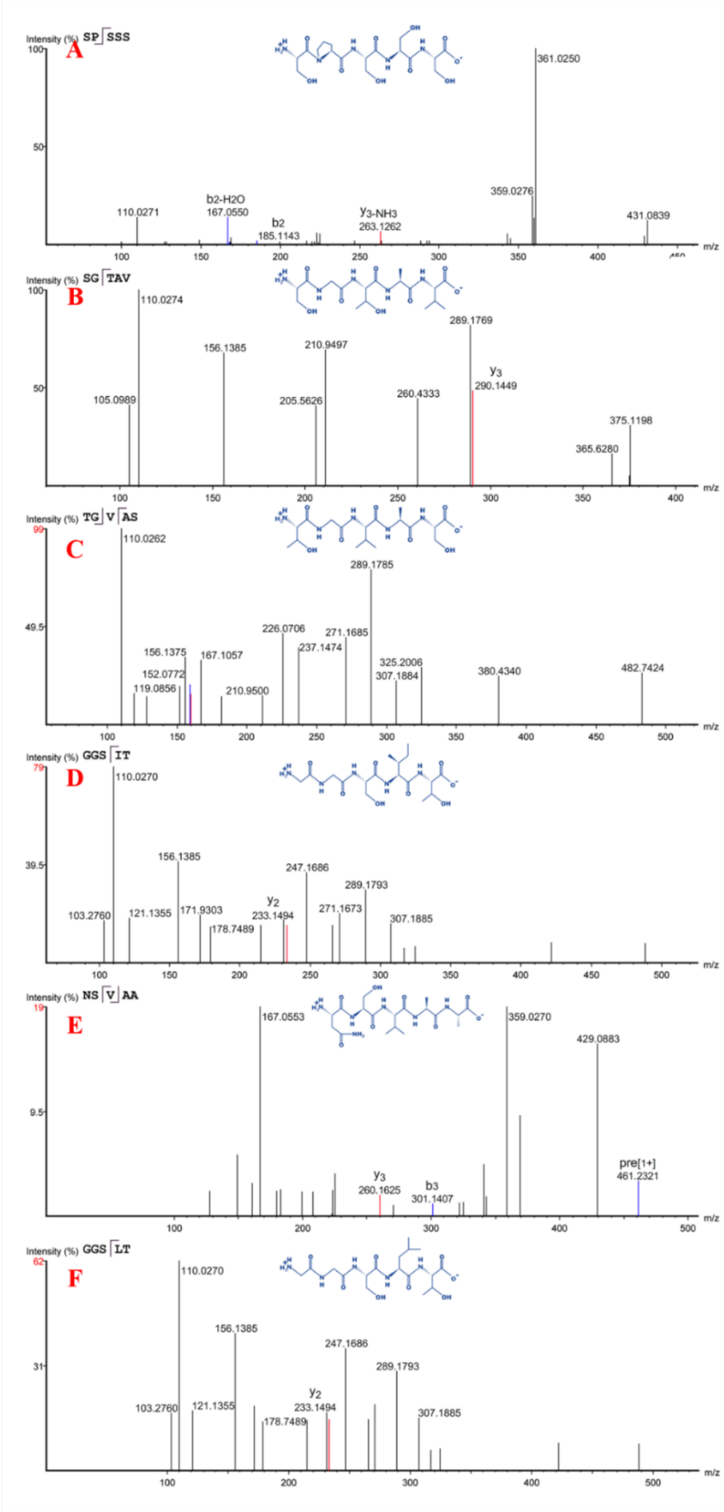

Figure S1. Secondary mapping of antioxidant peptides in proteomics. (A) SPSSS; (B) STGAV; (C) TGVAS; (D) GGSIT; (E) NSVAA; (F) GGSLT.

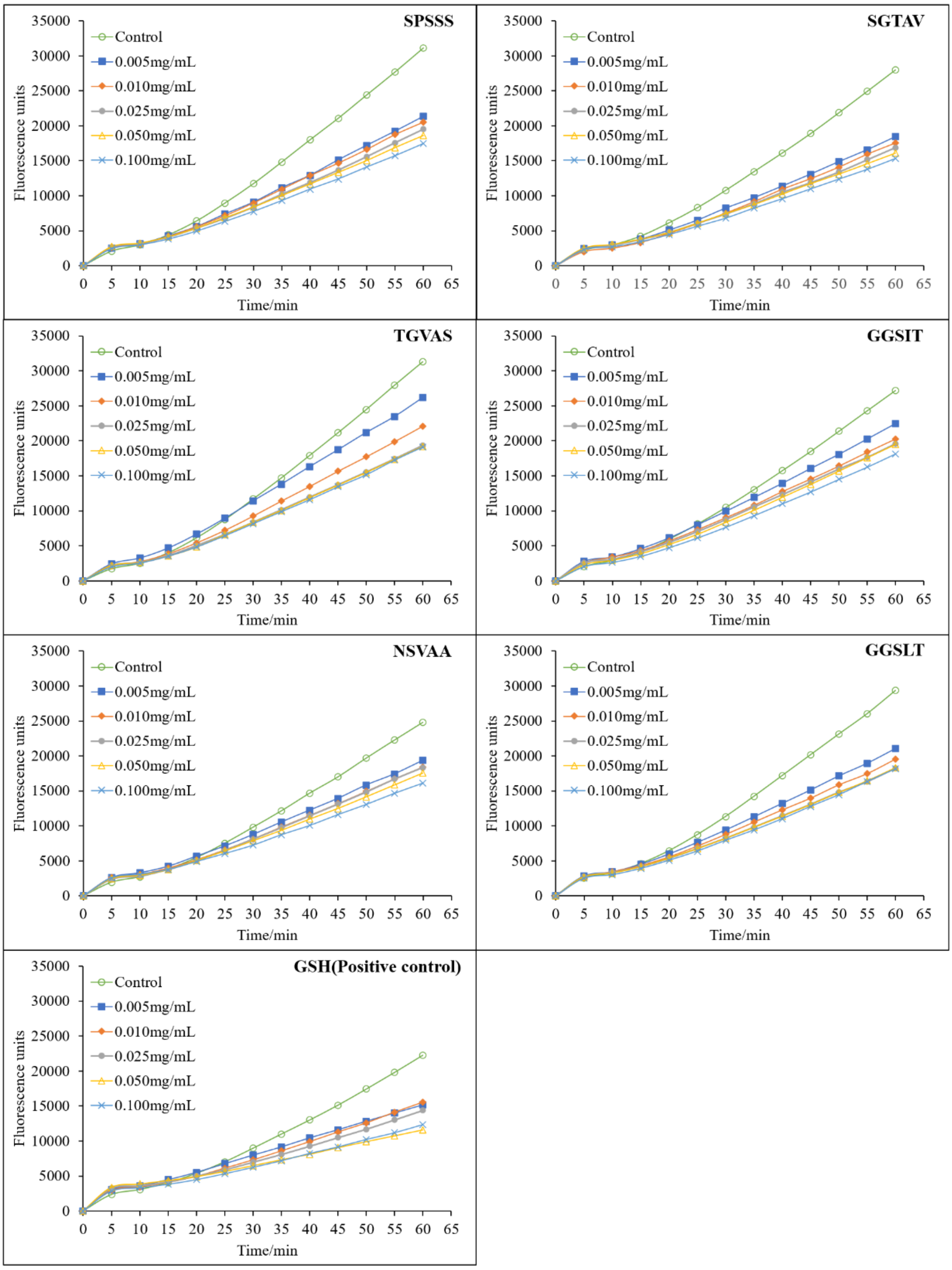

Figure S2. Curve of the effect of antioxidant peptides SPSSS, SGTAV, TGVAS, GGSIT, NSVAA, GGSLT and positive control (GSH) on probe fluorescence intensity of HepG2 cells in CAA.
